# The HIT-6 Questionnaire Corresponds to the PedMIDAS for Assessment of Pediatric Headaches

**DOI:** 10.3390/healthcare13233158

**Published:** 2025-12-03

**Authors:** Jacob Genizi, Raneen Mansour, Malak Burbara, Shoshana Gal, Keren Nathan, Lisa Kaly, Liat Yaniv

**Affiliations:** 1 Pediatric Department, Bnai Zion Medical Center, Haifa 3104802, Israel; 2 Rappaport Faculty of Medicine, Technion—Israel Institute of Technology, Haifa 3525422, Israel; 3 The Rheumatology Unit, Bnai Zion Medical Center, Haifa 3104802, Israel

**Keywords:** PedMidas, HIT-6, children, headache, migraine

## Abstract

**Objective:** The aim of our study was to compare two questionnaires regarding their ability to globally assess the impact of headaches on daily functioning in children as a primary endpoint and, secondarily, to evaluate their correlation to frequency and headache strength. **Background:** Headache is a common complaint in children and adolescents, leading to functional impairment. The impact of primary headaches, such as migraine and tension-type headaches, varies according to pain severity and frequency. Although the PedMIDAS questionnaire is a validated tool for assessing headache-related impact in children, it can be difficult for children to complete. The HIT-6 questionnaire is user-friendly but has been validated exclusively for use in adults. **Methods:** Our method involved a prospective cohort study in children aged 6–18 years who visited the headache clinic at Bnai Zion Medical Center due to primary headaches. All children filled in both the PedMIDAS and HIT-6. Data on headache diagnosis, frequency and intensity along with demographic data were obtained. **Results:** Of the 100 children participating, 96 completed both questionnaires. The final sample was 66% (63) female, and the average age was 14 years (±3.3). Migraine was reported by 62% (60), followed by tension-type headaches (18%) and mixed headache (15%). A weak positive spearman correlation was observed between PedMIDAS and HIT-6 scores to age (respectively, ρ 0.3 with *p* value < 0.005, and ρ 0.2 with *p* value < 0.05), a weak positive spearman correlation as well between the HIT-6 score and both disease duration and headache intensity (respectively, ρ 0.221 with *p* value < 0.05 and ρ 0.250 with *p* value < 0.05). PedMIDAS score was weakly positively correlated to headache frequency (ρ 0.27 with *p* value < 0.05). A moderately positive spearman correlation was found between the PedMIDAS and HIT scores with ρ 0.6 and *p* value < 0.005. Linear regression analysis revealed a stronger correlation with headache frequency for the HIT-6 than for the PedMIDAS, when adjusted to gender and headache type. **Conclusions:** The HIT-6 questionnaire correlates with the PedMIDAS questionnaire and can serve as a good alternative for easily evaluating headache burden in children.

## 1. Introduction

Migraine headaches may affect up to 10.6% of children aged 5 to 15 and 28% of adolescents aged 15 to 19 [1,2]. The burden of headache disorders in children and adolescents differs significantly from that in adults, both in how the headaches manifest and in their impact on daily life [3,4,5]. In children and adolescents, headaches often lead to frequent school absences, missed lessons, and decreased academic performance, which can obstruct learning and disrupt the child’s educational development [6]. Additionally, headaches can interfere with participation in sports, hobbies, and social activities, leading to feelings of isolation and impacting the formation of peer relationships during a critical period of social and emotional development [7]. Notably, while headaches in adults also affect social interactions, adults generally have more autonomy to manage their condition and make lifestyle adjustments, whereas children are more dependent on caregivers and face unique challenges in navigating school and social environments. In addition, younger children may struggle to articulate their symptoms, making diagnosis more challenging, while adolescents may experience anxiety or depression as a consequence of chronic headaches. All of these problems can result in altered family dynamics, with parents often needing to adjust their schedules or routines to manage the child’s condition [8].

The PedMIDAS (Pediatric Migraine Disability Assessment) questionnaire [9,10], adapted from the MIDAS (Migraine Disability Assessment) questionnaire used in adults [11,12], is currently the only validated tool for evaluating the burden of headaches in children and adolescents. Just as the MIDAS quantifies the impact of headaches in adults by tallying, e.g., the number of work days missed due to headaches over a three-month period, the PedMIDAS provides a structured measure of headaches’ impact on children’s daily functioning by quantifying headache-related disruptions in school attendance, home activities, sports, and social interactions over the previous three months. By translating headache burden into a numerical score, the PedMIDAS offers clinicians an objective method to gauge the severity and frequency of headaches, enabling better tracking of disease progression and treatment efficacy. Its standardized approach facilitates communication between healthcare providers, parents, and patients, aiding in tailored treatment plans. For these and other reasons, the PedMIDAS is considered essential for comprehensive headache management in pediatric populations [11]. However, younger children may face challenges with recall over a three-month period, potentially affecting the accuracy of the results. In addition, the focus on quantifiable measures like missed school days potentially risk missing the broader impact of headaches on patients’ well-being.

The HIT-6 questionnaire [13,14] offers a broad assessment of headache-related disability in adults and is increasingly recognized for its practicality in evaluating headache burden compared to the MIDAS questionnaire. While the MIDAS focuses on quantifiable measures (e.g., work days missed), the HIT-6 provides a more comprehensive view by assessing six key areas: pain severity, social functioning, work-related impact, role limitations, cognitive interference, and psychological distress. This makes the HIT-6 more versatile, able to capture the multifaceted impact of headaches on daily life. Additionally, the HIT-6 is simpler and faster to complete, requiring less recall, which reduces the risk of inaccuracies, especially in populations with memory challenges. For these reasons, many practitioners regard the MIDAS and the HIT-6 as complementary tools, with the former offering a more precise measurement of disability and the latter a qualitative overview [14].

Although the HIT-6 is validated only for adults, some researchers have used it for assessing children [7], and Piebes et al. reported moderate test–retest reliability for the HIT-6 and PedMIDAS in healthy adolescent athletes [15]. The aim of the present study was to compare the PedMIDAS and HIT-6 questionnaires in their ability to assess the impact of headaches in a pediatric population.

## 2. Patients and Methods

We conducted a prospective cohort study. Children aged 6–18 years who visited the headache clinic at Bnai Zion Medical Center due to primary headaches were enrolled consecutively to the study between January 2023 to December 2023. We did not estimate a sample size former to the study design elaboration. After their caregivers signed informed consent, all children filled in both the PedMIDAS and HIT-6 questionnaires. The two questionnaires were provided at the same time, with no instructions to the staff as to their arrangement on the clipboard, and no instructions to patients on the order in which they should be completed. A single researcher (J.G.) administered all the questionnaires. Separately, data was obtained on the children’s headache diagnosis, frequency and intensity, along with demographic data. Frequency was measured as number of episodes per week and VAS intensity scale from 1 (least severe) to 10.

The PedMIDAS (Pediatric Migraine Disability Assessment Scale) questionnaire is a tool used to assess the impact of headaches in children and adolescents aged 4–18 years. It helps medical staff understand the degree to which headaches affect a child’s daily life, including their school attendance, sports activities, homework, and social interactions. The scale consists of six questions eliciting the number of days over the past three months that the child missed or performed poorly at school, home activities, or other daily tasks due to headaches. Each answer is scored, and these scores are then summed to provide a total score reflecting the severity of the headaches’ impact on the child’s life [9]. Total scores are converted into a grade or level, with scores of 10 or less defined as level 1 (least impact on daily functioning), and scores of 50 or above as level 4 (most severe impact). [11]. See Supplementary File S1.

The HIT-6 (Headache Impact Test) questionnaire is a simple tool used to assess headaches affecting a person’s daily life. It is commonly used for adults and covers the impact of headaches on work, daily activities, social functioning, and overall quality of life. Six questions focus on different aspects on how headaches interfere with everyday activities (e.g., by causing severe pain, affecting concentration, causing tiredness, or impacting mood). For each of the six items, a 5-point scale from “never” to “always” measures how often their headaches affected their functioning over the past four weeks. Again, as with the PedMIDAS, the score is converted into a level, with scores of 36–49 reflecting little or no impact (level 1), and 60–78 reflecting the most severe impact (level 4). The HIT-6 is quick and easy to complete, making it a useful tool for tracking headache severity and monitoring treatment effectiveness over time [13]; see Supplementary File S2.

Descriptive statistics were used to summarize the data. Chi-squared test was used to compare categorical variables in independent groups, and Fisher’s exact test was used when expected frequency of one or more cells was less than 5. Student’s *t*-test was used to compare continuous variables or Mann–Whitney U test in case of non-normal distribution of the variable. Kruskal–Wallis test was used to compare more than two independent continuous variables. The Spearman correlation test was used for the correlation of continuous non-normal distributed variables. For non-monotonic, non-linear correlation between continuous variables, we run a generalized additive model (GAM). The nature of the correlation was graphically analyzed (smooth curve). Confounders were selected based on the author knowledge and the former univariate analysis. Multivariate analysis was performed with linear regression (ANCOVA). If the assumptions of the linear were not respected, we proceed for a Logarithmic, square root or power transformation, depending on the needs and the data. In all analyses, a two-tailed *p* value of 0.05 was used as the cut-off point for statistical significance. All the statistical analyses were performed with R Core Team (2023). R: A Language and Environment for Statistical Computing. R Foundation for Statistical Computing, Vienna, Austria.

The study was approved by the local IRB, # BNZ187-22.

## 3. Results

Demographic data are reported in Table 1. Of the 100 children enrolled in the study, 96 completed both questionnaires. Of these, 66% (63) were female, and the average group age was 14 years (±3.3). The most common headache diagnosis was migraine, reported by 62% (60) of the participants. Another 18% (17) had tension-type headache (TTH) according to the International Classification of Headache Disorders (ICHD)-3 criteria [16], and 15% (14) reported mixed headache (combined migraine and TTH). Data by diagnosis are presented in Table 2. Females dominated all the main diagnosis categories, most strikingly in the mixed headache group. Family history of headache was common in most groups (56–77%), with no difference between migraine and TTH. Headaches had occurred for an average of 11 months, with the longest duration in the migraine with aura group (15.4 months), and the shortest in the TTH group (8.1 months). Children with migraine had more intense headache attacks (6.71–7.67 on a scale of 1–10) compared to TTH (5.53), but their headache frequency was lower (3/week vs. 4.75/week for migraine and TTH, respectively).

**Figure 1:**
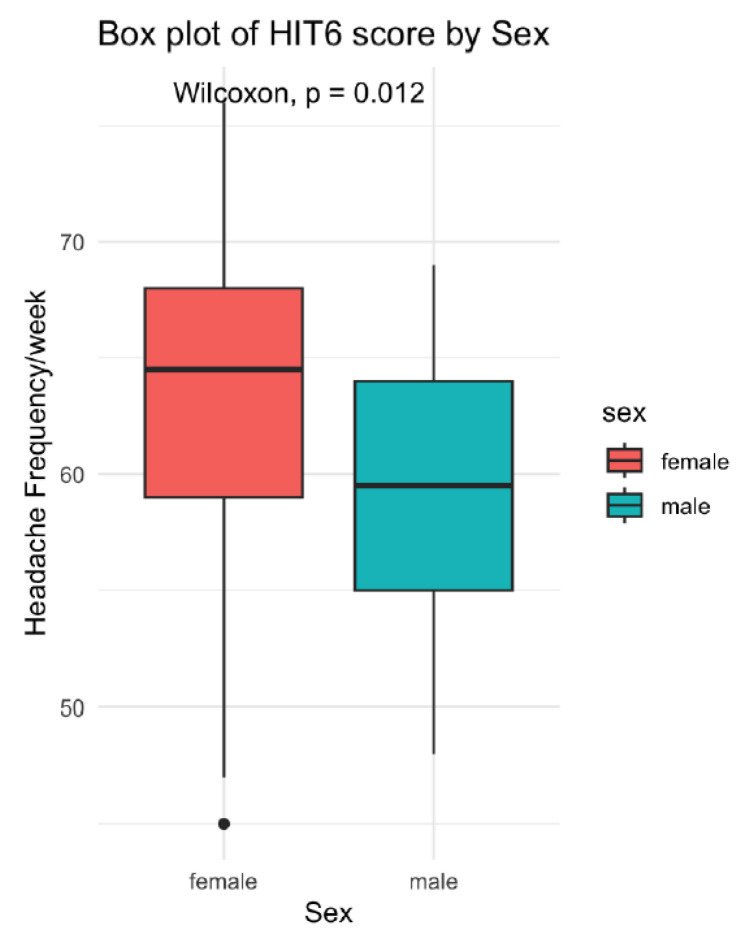
Headache frequency and gender difference with regard to the HIT-6 scores.

Children with migraine scored significantly higher on both questionnaires compared to children with TTH. For the PedMIDAS, the average level (computed from total scores) was 2.56–2.80 for the migraine group, and 1.71 for children with TTH. For the HIT-6, these figures were 3.44–3.90 for the migraine group, and 2.47 for TTH (Table 3). In addition, females had statistically significant higher HIT-6 scores compared to males (Figure 1).

Univariate analysis was used to examine the correlations between the two headache evaluation questionnaires and continuous variables. Both the PedMIDAS and HIT-6 levels correlated with headache diagnosis (*p* = 0.008–0.0006). PedMIDAS levels and total scores showed positive and significant but weak correlations with both age and headache frequency. HIT-6 levels showed positive and significant but weak correlations with age and headache strength. HIT-6 total scores showed positive and significant but weak correlations with age, disease duration, and headache strength (Table 4).

**Figure 2:**
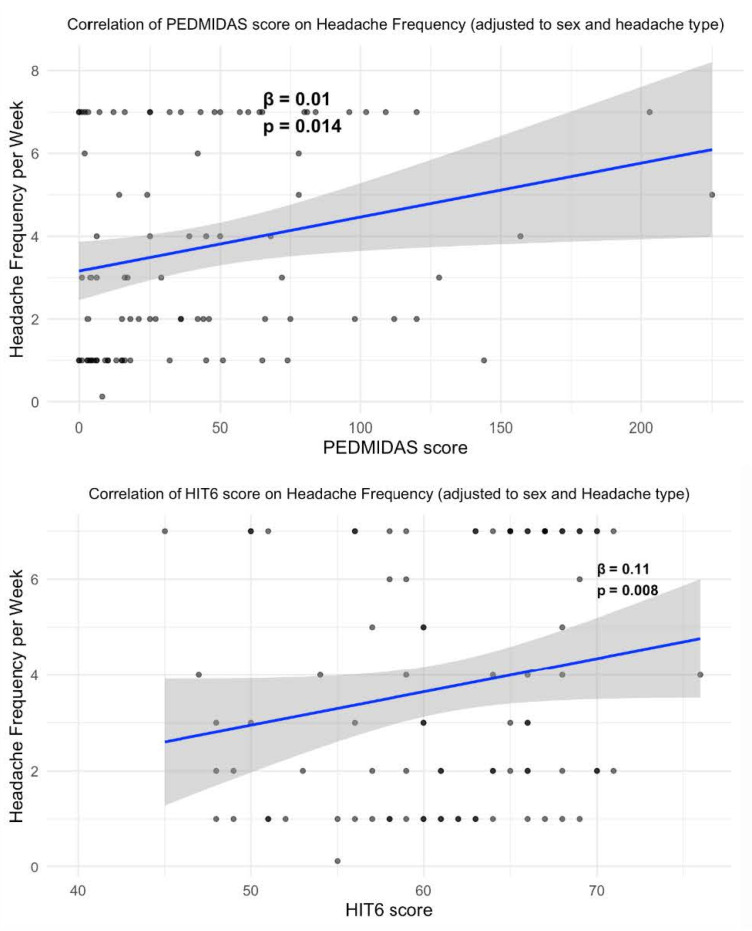
Correlation of PedMIDAS and HIT-6 scores with headache frequency. The model includes gender and headache type.

A linear regression model showed that the HIT-6 correlated with headache frequency even better than the PedMIDAS (Figure 2).

## 4. Discussion

Currently, the PedMIDAS questionnaire is the only tool validated for assessing the impact of headache in children. While the HIT-6 questionnaire is validated only for adults, its advantages compared with the PedMIDAS have led some to use it as a complementary tool [14]. Our findings show that the HIT-6 in children is correlated with the PedMIDAS.

In our study, PedMIDAS levels and scores were higher in migraine patients, with even higher scores observed in those with aura, compared to patients with TTH (*p* = 0.006). This finding aligns with previous research by Ozge [17] and Kröner-Herwig [18], though those earlier studies did not distinguish between migraine with and without aura. Notably, we observed the same pattern with the HIT-6 questionnaire, with significantly higher scores among migraine patients, particularly those with aura, compared to TTH patients (*p* < 0.001). With respect to the findings on aura, the present findings stand in contrast with recent work in adults [19] and a previous study in children [20], which found no difference in headache presentation in patients suffering from migraine with vs. without aura.

In our study, PedMIDAS scores correlated with headache frequency but not with headache severity. Similarly, Kröner-Herwig et al. [18] identified frequency as a stronger predictor than headache intensity. Ozge et al. [17] also reported a significant relationship between PedMIDAS scores and headache frequency, along with a weak correlation between the PedMIDAS and headache severity. In contrast, the HIT-6 questionnaire in our study was found to correlate with headache intensity better than with headache frequency.

Comparing the two questionnaires, the primary difference is their time frame: the PedMIDAS assesses the past three months, while the HIT-6 focuses on the past four weeks. Evaluating headache burden over an extended period provides a more comprehensive understanding of how the condition may vary over time, offering valuable insights into patterns and triggers that may not be apparent with shorter assessments. However, the use of retrospective headache diaries, particularly in children, introduces challenges. Children may struggle with accurately recalling past headache episodes, leading to incomplete or inaccurate records. This recall bias can undermine the reliability of the data, potentially affecting both clinical assessments and research outcomes. Thus, while a longer time frame may enhance perspective, it also increases the risk of misreporting, especially in younger populations.

The PedMIDAS and HIT-6 questionnaires also differ in their approach to assessing headache impact. The PedMIDAS measures the number of days a child’s activities are affected by headaches over three months, providing an objective assessment of how frequently headaches disrupt daily life. This includes missed school days or reduced participation in usual activities. In contrast, the HIT-6 evaluates the overall personal impact of headaches, assessing factors like pain intensity, emotional distress, social functioning, and interference with daily tasks over the past four weeks. Thus, while the PedMIDAS focuses on the extent of disability in terms of days affected, the HIT-6 captures the broader subjective experience of headache impact.

A related concern with the PedMIDAS relates to timing effects. Heyer et al. [21] reported that disability scores recorded during the school year differed from those recorded during summer holidays. They explained that this discrepancy reflects a ceiling effect associated with the PedMIDAS instrument. Although both the PedMIDAS and MIDAS measure similar types of disability, adults generally face fewer disruptions in their work or household activities during extended summer holidays, making the impact on MIDAS scores less pronounced. Weekends and shorter holidays tend to balance out over the three-month recall period for adults. As a result, if headache burden remains constant, MIDAS scores are likely to stay relatively stable year-round [22]. In contrast, PedMIDAS scores taken midway through the school year might be significantly higher than those collected at the end of summer, even with similar headache frequency and intensity. This seasonal variation could cause false positive or false negative treatment effects in clinical trials using the PedMIDAS as an outcome measure. As discussed earlier, the HIT-6 questionnaire assesses the overall impact of headaches on a patient’s well-being rather than focusing on missed school days. This broader perspective makes the HIT-6 less affected by vacation periods, providing a more stable measure of headache impact.

Finally, the more nuanced picture offered by the HIT-6 may have implications with regard to gender. In our study, there were no differences in PedMIDAS scores between males and females, consistent with the findings of Ozge et al. [16], though not those of Kröner-Herwig et al. [17], who found gender but not age differences. However, when examining the HIT-6 questionnaire, we observed that females had notably higher scores compared to males, indicating a greater perceived impact of headaches on their daily lives. This suggests that while the PedMIDAS may not always capture gender-related differences in headache impact, the HIT-6 might be more sensitive to these variations, particularly in assessing the personal burden of headaches among females.

## 5. Limitations

Our study was a single-center investigation involving a limited number of children treated at a tertiary headache clinic. To strengthen the validity of our findings, additional research with larger epidemiological cohorts is necessary. Another important limitation is that no a priori sample size calculation or power analysis was performed. This represents an important methodological limitation, as it raises concerns about whether the study was adequately powered to detect meaningful differences or draw robust conclusions. The absence of such analysis may affect the reliability and generalizability of the findings. Future studies should incorporate a formal power calculation to ensure sufficient sample size and methodological rigor. A further limitation is the fixed order in which the questionnaires were administered, with participants completing the PedMIDAS before the HIT-6. The lack of randomization may have introduced an order effect, potentially shaping how respondents answered the second questionnaire.

## 6. Conclusions

In our study, the HIT-6 questionnaire showed correlation with the PedMIDAS questionnaire, suggesting its potential as a valuable alternative for assessing headache burden in children, or at minimum a complementary tool. While the PedMIDAS specifically measures the number of days impacted by headaches, HIT-6 offers a broader evaluation of headache-related disability by assessing pain intensity, emotional well-being, and the overall impact on daily activities. Given that the HIT-6 is shorter, easier to administer, and applicable to a wide range of headache types, it may be particularly useful in clinical settings affected by time constraints.

## Figures and Tables

**Table 1:** Demographic data.

Characteristic	N = 96
**Age**	14.0 (±3.3)
**Sex**	
**Female**	63 (66%)
**Male**	33 (34%)
**Headache type**	
**Migraine with aura**	10 (10%)
**Migraine without aura**	50 (52%)
**TTH**	17 (18%)
**Combined**	14 (15%)
**Other**	5 (5.2%)
**Family history of migraine**	
**Negative**	32 (34%)
**Positive**	61 (66%)
**Disease duration (months)**	11.0 (9.0, 15.0)
**Headache frequency (per week)**	3.00 (1.00, 7.00)
**Headache intensity**	7.00 (5.00, 8.00)

**Table 2:** Headache data and diagnosis.

Characteristic	Migraine with Aura, N = 10 ^1^	Migraine Without Aura, N = 50 ^1^	TTH, N = 17 ^1^	Combined, N = 14 ^1^	Other, N = 5 ^1^	*p* Value
**Age**	14.9 (2.8)	13.0 (3.3)	11.7 (3.3)	14.8 (2.9)	12.8 (2.6)	**0.048**
**Sex**						0.12
**Female**	6/10 (60%)	32/50 (64%)	10/17 (59%)	13/14 (93%)	2/5 (40%)	
**Male**	4/10 (40%)	18/50 (36%)	7/17 (41%)	1/14 (7.1%)	3/5 (60%)	
**Family history of migraine**						0.7
**Negative**	4/9 (44%)	16/50 (32%)	7/17 (41%)	3/13 (23%)	2/4 (50%)	
**Positive**	5/9 (56%)	34/50 (68%)	10/17 (59%)	10/13 (77%)	2/4 (50%)	
**Disease duration (months)**	15.4 (4.9)	10.8 (4.3)	8.1 (5.0)	12.0 (5.0)	11.0 (3.9)	**0.004**
**Headache frequency (per week)**	3.00 (2.56)	3.04 (2.27)	4.75 (2.52)	5.43 (2.10)	3.38 (3.45)	**0.006**
**Headache intensity**	7.67 (0.87)	6.71 (1.50)	5.53 (1.19)	6.62 (1.45)	6.00 (1.00)	**0.005**

^1^ Mean (SD); n/N (%).

**Table 3:** Headache questionnaire results (total scores and levels) by headache diagnosis.

	Migraine with Aura	Migraine Without Aura	TTH	Combined	Other	*p* Value
**PedMIDAS score**	69 (69)	44 (47)	19 (26)	59 (36)	48 (83)	**0.006**
**PedMIDAS level**	2.80 (1.40)	2.56 (1.21)	1.71 (0.99)	3.21 (0.80)	2.00 (1.41)	**0.008**
**HIT-6 score**	65 (4)	62 (7)	55 (6)	63 (5)	54 (10)	**<0.001**
**HIT-6 level**	3.90 (0.32)	3.44 (0.95)	2.47 (1.18)	3.64 (0.50)	2.40 (1.14)	**<0.001**

**Table 4:** Correlations between headache evaluation questionnaires (total scores and levels) and continuous variables.

	ID	Age (Years)	Disease Duration (Months)	Headache Frequency (per Week)	Headache Intensity	PedMIDAS Score	PedMIDAS Level	HIT-6 Score
**ID**								
**Age (years)**	−0.317 **							
**Disease duration (months)**	0.011	0.182						
**Headache frequency (per week)**	−0.196	0.150	−0.142					
**Headache intensity**	0.107	0.219 *	0.269 *	−0.188				
**PedMIDAS score**	−0.245 *	0.326 **	0.150	0.275 *	0.075			
**PedMIDAS level**	−0.280 **	0.335 **	0.094	0.299 **	0.059	0.964 ***		
**HIT-6 score**	−0.031	0.244 *	0.225 *	0.203	0.255 *	0.594 ***	0.601 ***	
**HIT-6 level**	−0.076	0.228 *	0.168	0.121	0.236 *	0.478 ***	0.498 ***	0.874 ***

* Modestly correlated; ** Moderately correlated; *** Highly correlated.

## Data Availability

The data presented in this study are available on request from the corresponding author. (please specify the reason for restriction, e.g., the data are not publicly available due to privacy or ethical restrictions).

## Supplementary Materials

The following supporting information can be downloaded at https://www.mdpi.com/article/10.3390/healthcare13233158/s1.

## Clinical Implications

While PedMIDAS remains the only validated tool for assessing headache-related disability in children, its susceptibility to recall bias and seasonal variation may limit accuracy in clinical practice. Incorporating HIT-6 alongside PedMIDAS could provide a more comprehensive assessment of headache burden, supporting more tailored management strategies for pediatric patients.
