# Supplementary material for: The HIT-6 Questionnaire Corresponds to the PedMIDAS for Assessment of Pediatric Headaches
Source: Healthcare (Basel). 2025 Dec 3;13(23):3158. doi: 10.3390/healthcare13233158 (PMC12692315; doi:10.3390/healthcare13233158)
Supplement: Supplementary file 1 [file healthcare-13-03158-s001.zip › Supplementary File S1.pdf]

MIDAS QUESTIONNAIRE
Migraine Disability Assessment

אנא ענה/י על השאלות הבאות המתייחסות לכאב הראש שלך במשך **3 החודשים האחרונים**.כתוב/י את התשובה במקום המתאים. יש לרשום את המספר אפס כאשר זו התשובה או אם לא ביצעת את הפעילות בשלושת החודשים האחרונים.

שאלה 1

בכמה ימים במשך 3 החודשים האחרונים לא הלכת לעבודה או לבית הספר בגלל כאב הראש שלך?

(אם אינך הולך/ת לעבודה או לבית הספר יש לרשום אפס)

ימים

שאלה 2

בכמה ימים במשך 3 החודשים האחרונים ירדה היעילות שלך בעבודה או בבית הספר במחצית, או יותר, בגלל כאב הראש שלך?

(אל תספור/י כאן את הימים אותם ספרת בשאלה מס' 1. אם אינך הולך/ת לעבודה או לבית הספר יש לרשום אפס)

ימים

שאלה 3

בכמה ימים במשך 3 החודשים האחרונים לא בצעת שיעורי בית או שחקת בגלל כאב הראש שלך?

ימים

שאלה 4

בכמה ימים במשך 3 החודשים האחרונים ירדה היעילות שלך בביצוע עבודות משק-בית במחצית, או יותר, בגלל כאב הראש שלך?

(אל תספור/י כאן את הימים אותם ספרת בשאלה מס' 3)

ימים

שאלה 5

בכמה ימים במשך 3 החודשים האחרונים החסרת פעילות משפחתית, חברתית או בילוי בגלל כאב הראש שלך?

סה"כ _____

עד כמה כאבי הראש מפריעים לך לתפקוד

בכלל לא 1 2 3 4 5 מאוד

PedMIDAS Grading Scale

The PedMIDAS grading scale is as follows:

PedMIDAS Score Range	Disability Grade
0 to 10	Little to none
11 to 30	Mild
31 to 50	Moderate
Greater than 50	Severe

Download the Tool

To download the pedMIDAS tool for research
