# Supplementary material for: The HIT-6 Questionnaire Corresponds to the PedMIDAS for Assessment of Pediatric Headaches
Source: Healthcare (Basel). 2025 Dec 3;13(23):3158. doi: 10.3390/healthcare13233158 (PMC12692315; doi:10.3390/healthcare13233158)
Supplement: Supplementary file 1 [file healthcare-13-03158-s001.zip › Supplementary File S2.pdf]

מדד להשפעת כאבי ראש HIT-6™

שאלון זה נכתב במטרה לסייע לך לתאר ולהסביר איך את/ה מרגיש/ה
ומה אינך יכול/ה לעשות בשל כאבי הראש שלך.

בכדי להשלים את השאלון, אנא סמן/י X בריבוע המתאים בכל שאלה.

1 כשיש לך כאבי ראש, באיזו תדירות הכאב הוא חמור?

☐ אף פעם ☐ לעתים רחוקות ☐ לפעמים ☐ לעתים קרובות ☐ תמיד

2 באיזו תדירות כאבי ראש מגבילים את יכולתך לבצע פעילויות יומיומיות רגילות,
כולל עבודות בית, עבודה, לימודים או פעילויות חברתיות?

☐ אף פעם ☐ לעתים רחוקות ☐ לפעמים ☐ לעתים קרובות ☐ תמיד

3 כשיש לך כאב ראש, באיזו תדירות את/ה מרגיש/ה שהיית רוצה לשכב לנוח?

☐ אף פעם ☐ לעתים רחוקות ☐ לפעמים ☐ לעתים קרובות ☐ תמיד

4 ב - 4 השבועות האחרונים, באיזו תדירות הרגשת עייף/ה מכדי לעבוד או לעשות פעילויות יומיומיות, בשל כאבי הראש שלך?

☐ אף פעם ☐ לעתים רחוקות ☐ לפעמים ☐ לעתים קרובות ☐ תמיד

5 ב - 4 השבועות האחרונים, באיזו תדירות התעצבנת או שנמאס לך מכאבי הראש שלך?

☐ אף פעם ☐ לעתים רחוקות ☐ לפעמים ☐ לעתים קרובות ☐ תמיד

6 ב - 4 השבועות האחרונים, באיזו תדירות כאבי ראש הגבילו את יכולתך להתרכז בעבודה או בפעילויות יומיומיות?

☐ אף פעם ☐ לעתים רחוקות ☐ לפעמים ☐ לעתים קרובות ☐ תמיד

טור 1	+	טור 2	+	טור 3	+	טור 4	+	טור 5
(6 נקודות לכל תשובה בטור זה)		(8 נקודות לכל תשובה בטור זה)		(10 נקודות לכל תשובה בטור זה)		(11 נקודות לכל תשובה בטור זה)		(13 נקודות לכל תשובה בטור זה)

בכדי לקבל ציון, סכום/סכמי את הניקוד עבור תשובותיך בכל טור.
אנא שתף/י את הרופא שלך בתוצאותיך במדד ה Hit-6 .

ציון סופי: ☐

ציונים גבוהים יותר מצביעים על
השפעה רבה יותר על חייך.
טווח הציונים האפשרי הוא 78- 36.

HIT-6™ Israel (Hebrew) Version 1.1
©2000, 2001 QualityMetric, Inc. and GlaxoSmithKline Group of Companies

(מבחן השפעת כאב-ראש)

מה אומרת התוצאה שלך?

אם התוצאה שלך 60 ומעלה

לכאבי הראש שלך ישנה השפעה חמורה ביותר על חייך. ייתכן שהנך סובל מכאב משתק ומתסמינים אחרים, קשים יותר מהתסמינים המוכרים לאנשים אחרים שסובלים מכאבי ראש. אל תניח לכאבי הראש שלך למנוע בעדך מליהנות מהדברים החשובים בחיים, כגון משפחה, עבודה או פעילויות חברתיות.

קבע **עוד היום** ביקור אצל רופא המשפחה שלך, כדי לדון עמו בתוצאה של מבחן HIT-6 ובכאבי הראש שלך.

אם התוצאה שלך 56 - 59

לכאבי הראש שלך ישנה השפעה משמעותית על חייך. כתוצאה מכך, ייתכן שהנך סובל מכאבים חמורים ומתסמינים נוספים שמונעים ממך, לעתים, לבלות עם משפחתך, בעבודה, בבית הספר או בפעילויות חברתיות.

קבע **עוד היום** ביקור אצל רופא המשפחה שלך, כדי לדון עמו בתוצאה של מבחן HIT-6 ובכאבי הראש שלך.

אם התוצאה שלך 50 - 55

נראה שיש לכאבי הראש שלך השפעה מסוימת על חייך. כאבי הראש אינם אמורים למנוע ממך לבלות עם משפחתך, בעבודה, בבית הספר או בפעילויות חברתיות.

אל תשכח לדון עם רופא המשפחה שלך בתוצאה של מבחן HIT-6 ובכאבי הראש שלך בפגישתכם הבאה.

אם התוצאה שלך 49 ומטה

נראה שאין לכאבי הראש שלך השפעה על חייך בשלב זה, או שהשפעתם מעטה. מומלץ לבחון את עצמך במבחן HIT-6 מידי חודש, על מנת להמשיך לעקוב אחר ההשפעה שיש לכאבי הראש שלך על חייך.

אם התוצאה שלך במבחן HIT-6 היא 50 ומעלה

עליך להציג את התוצאות בפני רופא המשפחה שלך. כאבי ראש שפוגעים באיכות חייך עלולים להיות מיגרנה.

בעת הביקור הבא אצל רופא המשפחה שלך, הצג לפניו את תוצאות מבחן HIT-6. המחקרים מוכיחים שכאשר הרופאים מבינים בדיוק עד כמה כאבי הראש משפיעים לרעה על חייהם של מטופליהם, הם נוטים יותר למצוא תוכנית טיפולית מוצלחת, שעשויה לכלול גם מתן תרופות.

את מבחן HIT ניתן למצוא גם באינטרנט בכתובת www.headachetest.com.

גרסת האינטרנט של המבחן מאפשרת לך להדפיס דו״ח אישי של התוצאות, וכן גרסה מפורטת מיוחדת עבור הרופא.

אל תשכח לבחון את עצמך שוב במבחן HIT-6, או לנסות את גרסת האינטרנט, על מנת להמשיך ולעקוב אחר התקדמותך.

אודות HIT

מבחן השפעת כאב ראש (HIT) הוא כלי עזר שמשמש למדידת ההשפעה של כאבי ראש על יכולת התפקוד במקום העבודה, בבית הספר, ובמפגשים חברתיים. התוצאה שלך במבחן מצביעה על השפעת כאב הראש על חיי היומיום ועל היכולת שלך לתפקד. מבחן HIT פותח על ידי צוות בינלאומי של מומחים לכאבי ראש מתחום הנוירולוגיה ורפואת המשפחה, בשיתוף עם המומחים לפסיכומטריה שפיתחו את הכלי להערכת המצב הבריאותי *SF-36®.

מבחן HIT אינו מיועד לספק ייעוץ רפואי בנוגע לדיאגנוזה או לטיפול רפואי. עליך לשוחח עם הרופא המטפל לקבלת ייעוץ ספציפי לגבי מצבך הרפואי.

SF-36® הוא סימן מסחרי רשום של Medical Outcomes Trust ושל John E. Ware, Jr.

©2001 QualityMetric, Inc. and GlaxoSmithKline Group of Companies. All rights reserved.
